# Establishment of a novel method to assess MEK1/2 inhibition in PBMCs for clinical drug development

**DOI:** 10.3389/fcell.2022.1063692

**Published:** 2022-12-12

**Authors:** Lara M. Schüssele, Julia Koch-Heier, Julian Volk, Markus W. Löffler, Katharina Hoffmann, Regina M. Bruyns, Oliver Planz

**Affiliations:** ^1^ Department of Immunology, Interfaculty Institute for Cell Biology, University of Tübingen, Tübingen, Germany; ^2^ Atriva Therapeutics GmbH, Tübingen, Germany; ^3^ Department of General, Visceral and Transplant Surgery, University Hospital Tübingen, Tübingen, Germany; ^4^ Department of Clinical Pharmacology, University Hospital Tübingen, Tübingen, Germany; ^5^ Cluster of Excellence iFIT (EXC 2180) “Image-Guided and Functionally Instructed Tumor Therapies”, University of Tübingen, Tübingen, Germany; ^6^ Nuvisan ICB (Part of Nuvisan), Neu-Ulm, Germany

**Keywords:** pharmacodynamics, zapnometinib, PBMCs, clinical drug development, MEK1/2 inhibitor

## Abstract

The Raf/MEK/ERK signaling pathway plays a key role in regulating cellular proliferation, differentiation, apoptosis, cytokine production, and immune responses. However, it is also involved in diseases such as cancer, and numerous viruses rely on an active Raf/MEK/ERK pathway for propagation. This pathway, and particularly MEK1/2, are therefore promising therapeutic targets. Assessment of target engagement is crucial to determine pharmacodynamics or the efficacy of a MEK1/2 inhibitor. In the field of infectious diseases, this is usually first determined in clinical trials with healthy volunteers. One method to detect MEK1/2 inhibitor target engagement is to assess the degree of ERK1/2 phosphorylation, as ERK1/2 is the only known substrate of MEK1/2. As healthy subjects, however, only feature a low baseline MEK1/2 activation and therefore low ERK1/2 phosphorylation in most tissues, assessing target engagement is challenging, and robust methods are urgently needed. We hence developed a method using PBMCs isolated from whole blood of healthy blood donors, followed by *ex vivo* treatment with the MEK1/2 inhibitor zapnometinib and stimulation with PMA to first inhibit and then induce MEK1/2 activation. As PMA cannot activate MEK1/2 upon MEK1/2 inhibition, MEK1/2 inhibition results in impaired MEK1/2 activation. In contrast, PMA stimulation without MEK1/2 inhibition results in high MEK1/2 activation. We demonstrated that, without MEK1/2 inhibitor treatment, MEK1/2 stimulation with PMA induces high MEK1/2 activation, which is clearly distinguishable from baseline MEK1/2 activation in human PBMCs. Furthermore, we showed that treatment with the MEK1/2 inhibitor zapnometinib maintains the MEK1/2 activation at approximately baseline level despite subsequent stimulation with PMA. As our protocol is easy to follow and preserves the cells in an *in vivo*-like condition throughout the whole handling process, this approach can be a major advance for the easy assessment of MEK1/2 inhibitor target engagement in healthy probands for clinical drug development.

## Introduction

The Raf/MEK/ERK signaling cascade is one of the crucial pathways for transmission of extracellular signals from neurotransmitters, growth factors, hormones, or other stimuli in the cell (Sebolt-Leopold, 2004; Meier et al., 2005; Yoon and Seger, 2006). Stimulation of extracellular receptors leads to Ras activation. Ras activates Raf, which then phosphorylates MEK1 and MEK2. Activated MEK1/2 subsequently activates ERK1 and ERK2 through phosphorylation (Sebolt-Leopold et al., 1999; Planz, 2013; Koch-Heier et al., 2022). ERK1/2 constitutes the only known substrate of MEK1/2 (Lorusso et al., 2005; Yoon and Seger, 2006; Koch-Heier et al., 2022) and eventually influences proliferation, differentiation, apoptosis and survival (Sebolt-Leopold, 2004; Lorusso et al., 2005; Meier et al., 2005; Yoon & Seger, 2006), but also cytokine production and cellular immune responses (Ludwig et al., 2003; Planz, 2013; Koch-Heier et al., 2022).

However, upon deregulation and constitutive activation, the Raf/MEK/ERK pathway is involved in the promotion and progression of different cancer types (Sebolt-Leopold, 2004; Lorusso et al., 2005; Meier et al., 2005; Jamieson et al., 2016), as well as other diseases such as lymphatic anomaly (Li et al., 2019). Furthermore, many viruses such as SARS corona viruses or Influenza A and B viruses, rely on Raf/MEK/ERK pathway activation for replication and propagation (Ludwig et al., 2003; Laure et al., 2020; Koch-Heier et al., 2022).

In this context, Raf/MEK/ERK pathway inhibition is a promising approach for anticancer drug development (Lorusso et al., 2005; Jamieson et al., 2016), antiviral intervention (Ludwig et al., 2003; Planz, 2013; Laure et al., 2020), and treatment of further diseases (Li et al., 2019). Especially MEK1/2 has evolved as a promising target due to its high substrate specificity (Sebolt-Leopold, 2004).

Since MEK inhibitors cover a broad range of potential applications, detecting MEK1/2 inhibitor binding to its target MEK1/2 (target engagement) is essential to assess pharmacodynamic (PD) parameters or efficacy. One method to detect MEK1/2 inhibitor target engagement is to assess the degree of ERK1/2 phosphorylation. Since ERK1/2 can only be phosphorylated by activated MEK1/2 (Yoon & Seger, 2006; Wortzel and Seger, 2011), MEK1/2 inhibition is directly linked to a decrease of ERK1/2 phosphorylation. This renders the degree of ERK1/2 phosphorylation a robust indicator for the MEK1/2 activation status. For instance, there are numerous reports about decreased ERK1/2 phosphorylation in cancer cells following MEK1/2 inhibitor treatment (McDaid et al., 2005; Solit et al., 2006; Ciuffreda et al., 2009; Iverson et al., 2009; Infante et al., 2012), as well as in virus infected cell and animal models (Pleschka et al., 2001; Laure et al., 2020; Koch-Heier et al., 2022; Schreiber et al., 2022).

However, assessing target engagement in clinical trials of infectious diseases is much more challenging than in cancer. In oncology studies, biopsy material with intrinsically high MEK1/2 activation can be obtained, which is usually impossible in clinical trials in infectious diseases. Although it is possible to assess such PD parameters in clinical trials with MEK1/2 inhibitors in other indications than cancer (Koch-Heier et al., 2022), low basal MEK1/2 activation in healthy adults remains an issue. This low basal MEK1/2 activation only allows a very limited dynamic range upon MEK1/2 inhibitor treatment. Therefore, robust methods to easily assess MEK1/2 inhibition as a PD parameter in clinical trials with MEK1/2 inhibitors in healthy participants are sought-after.

We therefore developed a method that could be used to assess MEK1/2 inhibition in peripheral blood mononuclear cells (PBMCs) of MEK1/2 inhibitor-treated healthy probands by *ex vivo* stimulation of the cells with phorbol myristate acetate (PMA). Following this approach in a clinical trial, PBMCs need to be isolated from whole blood, which is sampled from MEK1/2 inhibitor-treated study participants at different time points. The cells will be maintained in autologous plasma, which is obtained during the isolation, and will be stimulated with PMA, which cannot activate MEK1/2 upon MEK1/2 inhibition. MEK1/2 inhibition therefore leads to impaired MEK1/2 activation. In contrast, PMA stimulation without MEK1/2 inhibition (as in a pre-dose control) results in increased MEK1/2 activation, which is shown by a high degree of ERK1/2 phosphorylation.

By *in vitro* proof of principle experiments of our method we demonstrated that, without MEK1/2 inhibitor treatment, MEK1/2 stimulation with PMA induces high MEK1/2 activation, which is clearly distinguishable from baseline MEK1/2 activation in human PBMCs. Simultaneously, we showed that treatment with the MEK1/2 inhibitor zapnometinib maintains the MEK1/2 activation near baseline levels despite subsequent stimulation with PMA. These results were reproducible across different analytic systems, which further supports the robustness of our method. Although our method was not yet applied *in vivo,* we assume that comparable results can be obtained upon *ex vivo* PMA stimulation of PBMCs isolated from MEK1/2 inhibitor-treated study participants in a clinical trial.

## Materials and equipment

### Laboratory equipment

• Automated cell counter (e.g., Cellometer Auto 2000 by Nexcelom; Cenibra GmbH, Bramsche, Germany)
• Cell culture incubator at 37°C, 5% CO_2_ (e.g., HERAcell 150i; Heraeus Instruments, Hanau, Germany)
• Centrifuge capable to hold 1.5 ml tubes and coolable (e.g., Biofuge fresco; Heraeus Instruments)
• Centrifuge with swinging bucket rotor capable to hold 50 ml tubes (e.g., Multifuge 3S-R; Heraeus Instruments)
• Heating block (e.g., Thermomixer comfort; Eppendorf, Hamburg, Germany)
• Laminar flow cell culture work bench (e.g., Tecnoflow 3F150-II GS; Integra Biosciences, Zizers, Switzerland)
• Manual pipettes, adjustable to various volumes (e.g., Discovery Comfort series; HTL, Warsaw, Poland)
• Microplate reader (e.g., SpectraMAX Plus 384; Molecular Devices, San José, CA, United States)
• Pipetting aid (e.g., Pipetboy acu; Integra Biosciences)
• Plate reader, capable of reading electrochemiluminescence (MESO Quickplex SQ 120; Meso Scale Discovery, Rockville, MD, United States)
• Thermo shaker (Thermo-shaker PHMP-4; GrantIinstruments, Cambridgeshire, United Kingdom)
• Vortex mixer (e.g., AnalogVortexMixer; Ohaus Corporation, NJ, United States)

### Consumables

• 1.5 ml tubes, sterilized by autoclaving (e.g., Eppendorf^®^ by Sigma-Aldrich, St. Louis, MO, United States)
• 50 ml centrifuge tubes, sterile (e.g., Greiner Bio-One, Kremsmuenster, Austria)
• 96-well flat-bottom cell culture plates (e.g., Greiner Bio-One)
• 96-well round-bottom cell culture plates (e.g., Greiner Bio-One)
• Butterfly needles, 21G (e.g., Safety-Multifly^®^ by Sarstedt, Nümbrecht, Germany)
• Hypodermic needles, 21G (e.g., BD Microlance™ 3 by BD Biosciences, Franklin Lakes, NJ, United States)
• Lithium heparin blood collection tubes (e.g., Greiner Bio-One)
• SepMate™-50 tubes (Stemcell Technologies, Vancouver, BC, Canada)
• Serological pipettes, sterile, various volumes (e.g., Falcon^®^ by Corning, Corning, NY, United States)
• Syringes, sterile, various volumes (e.g., BD Biosciences)
• Pipette tips, sterilized by autoclaving, various volumes (e.g., Greiner Bio-One)

### Reagents

• Benzonase^®^ Nuclease (Sigma-Aldrich)
• Dimethyl sulfoxide (DMSO) (Merck, Darmstadt, Germany)
• Ethylenediaminetetraacetic acid (EDTA) (Sigma-Aldrich)
• Glycerol (Carl Roth, Karlsruhe, Germany)
• Lipopolysaccharide (LPS) from *E. coli* O55:B5 (Sigma-Aldrich)
• Lymphoprep™ (Stemcell Technologies)
• Phenylmethylsulphonyl fluoride (PMSF) (Sigma-Aldrich)
• Phosphatase inhibitor cocktail (Roche, Basel, Switzerland)
• Phosphate buffered saline (PBS) (Gibco, Carlsbad, CA, United States)
• PMA (Sigma-Aldrich)
• Protease inhibitor cocktail (Roche)
• Sodium chloride (NaCl) (Carl Roth)
• Sodium deoxycholate (Sigma-Aldrich)
• Sodium dodecyl sulfate (SDS) (Carl Roth or Gibco)
• Sodium heparin (10.000 I.E./ml) (Braun, Melsungen, Germany)
• Tris base (Sigma-Aldrich)
• Triton X-100 (Sigma-Aldrich)
• Tumor necrosis factor alpha (TNF-α) (Sigma-Aldrich)
• Zapnometinib (ChemCon GmbH, Freiburg im Breisgau, Germany)

### Kits used in the context of Wes™ analysis

• Anti-rabbit detection module (ProteinSimple^®^, San José, CA, United States)
• EZ Standard Pack 1 (12–230 kDa) (ProteinSimple^®^)
• Jess/Wes Separation Kit (12–230 kDa) (ProteinSimple^®^)

### Kits used in the context of MSD analysis

• Inhibitor pack (Meso Scale Discovery)
• Phospho/Total ERK1/2 Whole Cell Lysate Kit (Meso Scale Discovery)

### Kits (other)

• CyQUANT™ LDH Cytotoxicity Assay Kit (Invitrogen, Waltham, MA, United States)

### Antibodies for Wes™ analysis

• ERK1/2: p44/42 MAPK (Erk1/2) (137F5) Rabbit mAb (Cell Signaling Technology, Danvers, MA, United States)
• pERK1/2: Phospho-p44/42 MAPK (Erk1/2) (Thr202/Tyr204) (D13.14.4E) XP^®^ Rabbit mAb (Cell Signaling Technology)

### Lysis buffer formulations

• 1x radio-immunoprecipitation assay (RIPA) buffer (used for sample preparation for the Wes™ system): 0.24% (w/v) tris base, 0.88% (w/v) NaCl, 0.2% (v/v) 500 mM EDTA, 1% (v/v) Triton X-100, 0.5% (w/v) sodium deoxycholate, 0.1% (w/v) SDS (Carl Roth), 10% (v/v) glycerol, 0.05% PMSF, 0.01% Benzonase^®^ Nuclease, protease inhibitor cocktail, and phosphatase inhibitor cocktail in water (ddH_2_O)
• Complete lysis buffer (used for sample preparation for the MSD system; use with the Wes™ system possible): Inhibitor pack Meso Scale Discovery, 2 mM PMSF Sigma-Aldrich, and 0.1% SDS (Gibco) in Tris lysis buffer (Meso Scale Discovery)

### Biological samples

Up to 50 ml whole blood for isolation of PBMCs were obtained from healthy adult volunteers (*N* = 11) registered with the Biobank of the Department of Immunology at the University of Tübingen (Project No. 156/2012BO1), after informed consent documented in writing. Ethical approval was obtained upon review by the Ethics Committee at the Medical Faculty of the Eberhard Karls University Tübingen and the University Hospital Tübingen (Project No. 887/2020BO2). The blood was drawn in sterile syringes. Sodium heparin was used as an anticoagulant. Furthermore, up to 40 ml whole blood for PBMC isolation were drawn from healthy adult volunteers (*N* = 5) who signed an informed consent form (ICF) from the Nuvisan GmbH for whole blood donation, as well as an informed consent form for the inclusion of Nuvisan employees in studies. The blood was drawn in lithium heparin blood collection tubes.

## Methods

### PBMC isolation from whole blood using SepMate™-50 tubes

15 ml Lymphoprep™ per tube were added to the bottom compartment of SepMate™-50 tubes. 10–12 ml whole blood of each donor were mixed in a 1:2.5 dilution with 15–18 ml PBS, and 25–30 ml of the mixture were added per SepMate™-50 tube to the top compartment of each tube. The tubes were centrifuged for 10 min (min) at 1,200 ×g at room temperature (RT). For the cytotoxicity assessment (see “*Cytotoxicity assessment*”) and the stimulation timeline (see “*Sample preparation procedure for stimulation timeline*”), 10–12 ml plasma (equal volume as the amount of PBS initially added to the whole blood) were removed from the cells to increase cell counts in the remaining plasma. The removed plasma was centrifuged for 10 min at 310 ×g to remove debris and residual cells and was set aside for later use in the cytotoxicity assay. The PBMCs and remaining plasma in the SepMate™-50 tubes were transferred to fresh 50 ml centrifuge tubes. Experiments were conducted immediately after PBMC isolation.

### Sample preparation procedure for stimuli comparison

PBMCs suspended in 30 ml autologous plasma obtained during the isolation were separated in six groups à 5 ml in 50 ml tubes. The cells in one tube each were treated with 1 μg/ml LPS from *E. coli* O55:B5 dissolved in PBS, 20 ng/ml TNF-α dissolved in PBS, or 400 nM PMA dissolved in DMSO, respectively. The cells in three control tubes were either left untreated (baseline control) or were supplied with PBS or DMSO (solvent controls). The PBS content was 2%, the DMSO content was 0.1%. The cells were incubated for 30 min at 37°C and 5% CO_2_ in a humidified cell culture incubator and the tubes were gently inverted two times every 10 min to prevent sedimentation of PBMCs. PBMCs were purified and lysed as described in the sections “*PBMC purification*” and “*Cell lysis using 1x RIPA buffer*.” Sample analysis was performed as described in the section “*Determination of the ERK1/2 phosphorylation status using the Wes™ technology*.”

### Sample preparation procedure for stimulation timeline

1 ml PBMC suspension in plasma was transferred to eleven 1.5 ml tubes each, to obtain 2 sets à 5 tubes and one single tube (baseline control). The cells in one set were treated with 400 nM PMA in 0.1% DMSO, the cells in the other set were supplied with 0.1% DMSO (DMSO control). The cells in the baseline control tube remained untreated. The tubes were incubated at 37°C while preventing cell sedimentation by gently inverting the tubes two times every 10 min. PBMC purification and cell lysis of one tube of each set was performed after 15, 30, 45, 60, and 120 min, or after 0 min for the baseline control. Therefore, the tubes were centrifuged for 5 min at 1,520 ×g and 4°C, the supernatant was discarded, and the cell pellets were washed with 1 ml ice cold PBS. Cell lysates were prepared as described in the section “*Cell lysis using 1x RIPA buffer*.” Sample analysis was performed as described in the section “*Determination of the ERK1/2 phosphorylation status using the Wes™ technology*.”

### Cytotoxicity assessment

Cytotoxicity of PMA in human PBMCs was assessed *via* Lactate dehydrogenase (LDH) release using the CyQUANT™ LDH Cytotoxicity Assay Kit following the manufacturer’s instructions. In short, 100 µl PBMC suspension in plasma were seeded per well in 96-well round-bottom cell culture plates. The cells were treated in quadruplicates with 3.13, 6.25, 12.5, 25, 50, 100, 200, 400, and 800 nM PMA dissolved in DMSO and diluted in autologous plasma to a DMSO concentration of 0.1%. Quadruplicate DMSO control wells were supplied with 0.1% DMSO in plasma, quadruplicate untreated (spontaneous LDH release) control wells were supplied with plasma. Treatment volume for all wells were 10 µl. 110 µl plasma were added to the plate in quadruplicate as a cell free “blank” control. 10 µl of 10x lysis buffer (provided with the kit) were added to one well of each quadruplicate set to lyse the cells as maximum LDH release control. The plates were incubated at 37°C and 5% CO_2_ in a humidified cell culture incubator for 45 min.

50 µl Reaction Mix (provided with the kit) were added per well to a 96-well flat-bottom cell culture plate and 50 µl plasma were transferred from each sample well in the round-bottom plate to a corresponding well in the flat-bottom plate. After 30 min incubation at RT in the dark, 50 µl Stop Solution (provided with the kit) were added per well and the absorbance was measured at 490 nm (primary wavelength) and 680 nm (reference wavelength).

The absorbance measured at 680 nm was subtracted from the absorbance at 490 nm and the cell viability was calculated according to the following formula:

$$\%\;Viability=100-\left(\frac{PMA\;treated\;LDH\;release\;–\;spontaneous\;LDH\;release}{Maximum\;LDH\;release\;–\;spontaneous\;LDH\;release}\right)∙100$$

### Sample preparation procedure for PMA titration

To assess the optimal PMA concentration required for stimulation, PBMCs suspended in autologous plasma in centrifuge tubes were treated with 25, 50, 100, 200, 400, or 800 nM PMA in 0.1% DMSO. A DMSO control was supplied with 0.1% DMSO, a baseline control was left untreated. After 30 min incubation at 37°C and 5% CO_2_ while preventing cell sedimentation by gently inverting the tubes two times every 10 min, PBMC purification and lysate preparation using 1x RIPA buffer was carried out as described in the sections “*PBMC purification*” and “*Cell lysis using 1x RIPA buffer*.” Sample analysis was performed as described in the section “*Determination of the ERK1/2 phosphorylation status using the Wes™ technology*.”

### MEK1/2 inhibition and stimulation in human PBMCs

PBMCs suspended in autologous plasma in centrifuge tubes were treated with 10, 25, or 50 μg/ml zapnometinib in 0.9% DMSO for 60 min at 37°C and 5% CO_2_ while preventing cell sedimentation by gently inverting the tubes two times every 10 min. A DMSO control was supplied with 0.9% DMSO, a positive and a baseline control remained untreated. Subsequently, the zapnometinib-treated cells as well as the DMSO and positive control cells were stimulated with 400 nM PMA for 30 min at 37°C and 5% CO_2_ while preventing cell sedimentation by gently inverting the tubes two times every 10 min. The baseline control remained unstimulated but was otherwise treated identical as the other cells. PBMC purification and lysate preparation using 1x RIPA buffer or Complete lysis buffer was carried out as described in the sections “*PBMC purification*,” “*Cell lysis using 1x RIPA buffer*,” and “*Cell lysis using Complete lysis buffer*.” Based on the experimental context, sample analysis was either performed as described in the sections “*Determination of the ERK1/2 phosphorylation status using the Wes™ technology*” (after cell lysis using 1x RIPA or Complete lysis buffer) or “*Determination of the ERK1/2 phosphorylation status using the Meso Scale Discovery system*” (after cell lysis using Complete lysis buffer).

### PBMC purification

For subsequent cell lysis using 1x RIPA buffer, PBS was added to each tube containing PBMCs in plasma to obtain a total volume of 50 ml. The tubes were centrifuged for 8 min at 800 ×g at RT, the supernatant was discarded, and the cell pellets were resuspended. 30 ml PBS were added to each pellet followed by 8 min centrifugation at 300 ×g. The supernatant was discarded completely before cell lysis.

For subsequent cell lysis using Complete lysis buffer, the cells were counted prior to the purification. PBS was then added to each tube containing PBMCs in plasma to obtain a total volume of 50 ml. The tubes were centrifuged for 3 min at 500 ×g at 4°C. The supernatant was discarded, and the cell pellets were resuspended. 1 ml ice-cold PBS was added to each pellet, the cells were transferred to 1.5 ml tubes and the tubes were centrifuged 3 min at 500 ×g at 4°C. The supernatant was discarded completely before cell lysis.

### Cell lysis using 1x RIPA buffer

This lysis protocol was followed prior to sample analysis with the Wes™ system. Ice cold 1x RIPA buffer was added to the cell pellets and the cells were resuspended in the buffer. Unless already performed, the cells in the buffer were transferred to separate 1.5 ml tubes. Lysis was performed for 15 min on ice. The lysates were centrifuged 5 min at 16,000 ×g and 4°C to remove cell debris, and the supernatants (lysates) were transferred to fresh 1.5 ml tubes. The lysates were stored at −80°C until analysis.

### Cell lysis using Complete lysis buffer

This lysis protocol was followed prior to sample analysis with the Meso Scale Discovery (MSD) system but can also be used for sample analysis with the Wes™ system. 100 µl Complete lysis buffer per 10^6^ PBMCs were added to the cell pellets and the cells were resuspended in the buffer. Lysis was performed for 15–30 min on ice. The lysates were centrifuged 10 min at ≥ 10,000 ×g at 4°C to remove cell debris, and the supernatants (lysates) were transferred to fresh 1.5 ml tubes. The lysates were stored at -80 °C until analysis.

### Determination of the ERK1/2 phosphorylation status using the Wes™ technology

To determine the ERK1/2 phosphorylation status using the Wes™ technology, the Jess/Wes Separation Kit (12–230 kDa), the EZ Standard Pack 1 (12–230 kDa), and the Anti-rabbit detection module were used according to the manufacturer’s instructions. In short, the 0.1x Sample Buffer, 400 mM dithiothreitol (DTT) solution, 5x Fluorescence Master Mix, and the Biotinylated Ladder were prepared following the manufacturer’s instructions. The lysates were diluted with 0.1x Sample Buffer, and 2 µl of the 5x Fluorescence Master Mix in DTT were added to 8 µl of diluted lysate. The samples were denatured for 5 min at 95°C and centrifuged for 5 min at 600 ×g. 1.8 µg protein were loaded onto the assay plate and analyzed using specific antibodies. The primary antibodies for phospho ERK1/2 (Phospho-p44/42 MAPK (Erk1/2) (Thr202/Tyr204) (D13.14.4E) XP^®^ Rabbit mAb, Cat# 4370) and total ERK1/2 (p44/42 MAPK (Erk1/2) (137F5) Rabbit mAb, Cat# 4695) detection were used at a 1:50 dilution in Antibody Diluent. The anti-rabbit secondary antibody (Cat# DM-001) was ready to use. Data were analyzed with Compass software for Simple Western (ProteinSimple^®^).

### Determination of the ERK1/2 phosphorylation status using the Meso Scale Discovery system

To determine the ERK1/2 phosphorylation status using the MSD system, the Phospho/Total ERK1/2 Whole Cell Lysate Kit was used according to the manufacturer’s instructions. In short, the multiplex assay consists of a plate (MULTI-SPOT 96-Well 4-Spot Phospho/Total ERK1/2 Plate) pre-coated with capture antibodies for phospho ERK1/2 (Thr202/Tyr204; Thr185/Tyr187) and total ERK1/2. The required number of plates was blocked (Blocking Solution) for a minimum of 1 h at RT on a thermo shaker shaking at 300–1,000 rpm, washed (Tris Wash Buffer), and the samples (PBMC lysates, 25 µl) were added in duplicate. After 3–3.5 h the plates were washed again and SULFO-tagged anti-total ERK1/2 antibody was added to the wells for 60–75 min. The plates were washed and Read buffer was added before analyzing the plates on the Meso QuickPlex SQ 120. The analysis of the data was based on ECL signals from the Meso Scale data capture and processing software Workbench 4.0.12.

### Stepwise procedure for a proof of principle experiment to show the effectiveness of a MEK1/2 inhibitor in PBMCs of healthy human adults

This stepwise procedure is divided in the sections “*PBMC isolation from whole blood using SepMate™-50 tubes*,” “*MEK1/2 inhibitor treatment*,” “*PMA stimulation*,” and “*PBMC purification*.”

**FIGURE 1:**
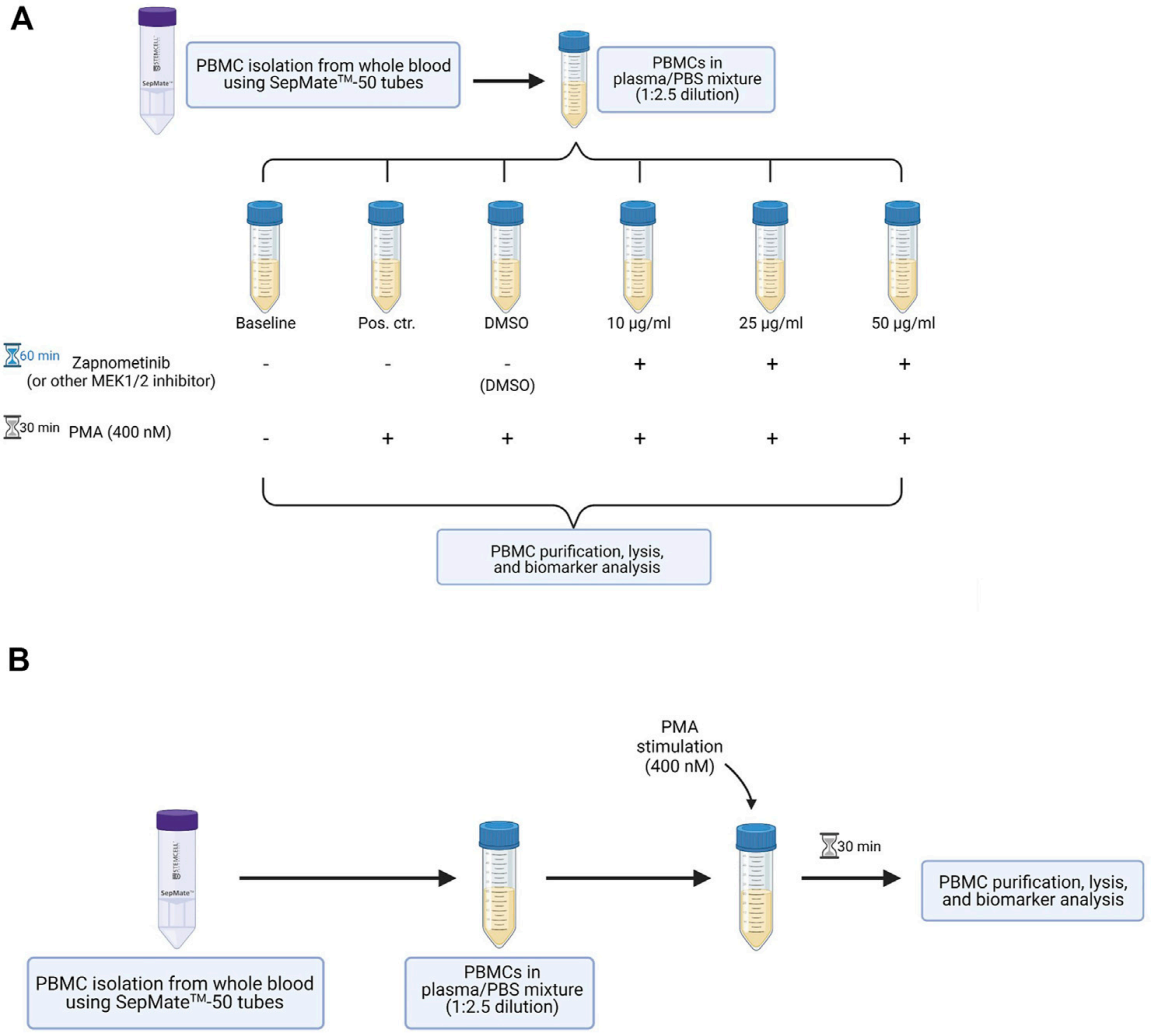
Overview of the method developed to assess MEK1/2 inhibition in PBMCs of healthy probands. **(A)** Workflow for the *in vitro* proof of principle experiment. PBMCs are isolated from whole blood and are maintained in the plasma obtained during PBMC isolation. The cells are treated with different concentrations of a MEK1/2 inhibitor for 60 min, followed by 30 min stimulation with 400 nM PMA. PBMCs are purified, lysed, and analyzed for their ERK1/2 phosphorylation status. **(B)** Workflow for the *in vitro* stimulation of PBMCs isolated from MEK1/2 inhibitor-treated subjects. PBMCs are isolated from whole blood and maintained in the plasma obtained during isolation. The cells are stimulated 30 min with 400 nM PMA, followed by purification, lysis, and analysis for their ERK1/2 phosphorylation status. This figure was created with BioRender.com.

It describes the different steps performed for sample preparation to assess the effectiveness of a MEK1/2 inhibitor by determining the ERK1/2 phosphorylation status in PBMCs of healthy human adults. The PBMCs can either be treated *in vitro* with a MEK1/2 inhibitor (Figure 1A) or can be isolated from whole blood of study participants treated with the MEK1/2 inhibitor (*in vivo* treatment, when clinical trial samples are available) (Figure 1B). If not noted otherwise, the steps described in the following apply for both scenarios. All steps are performed under aseptic/sterile conditions.

#### PBMC isolation from whole blood using SepMate™-50 tubes

(1) Determine the number of SepMate™-50 tubes needed for the isolation. One SepMate™-50 tube is required per 10–12 ml whole blood.
(2) Transfer 15 ml Lymphoprep™ to each SepMate™-50 tube.
  (a) Place the outlet of the pipette filled with Lymphoprep™ directly onto the central hole in the insert in the SepMate™-50 tube.
  (b) Carefully and slowly pipette the Lymphoprep™ through the central hole.
  (c) As soon as the Lymphoprep™ level rises above the insert, remove the serological pipette from the central hole and add the remaining Lymphoprep™ on top.

Note: This step can be done up to 2 hours before the PBMC isolation. Keep the SepMate™-50 tubes filled with Lymphoprep™ at RT in the dark until use.

(3) Draw whole blood from a healthy volunteer/study participant.

Note: Use an anticoagulant to prevent coagulation. For the here established method only heparin was used. The effect of another anticoagulant on the outcome of the results is therefore not known and needs to be assessed by the user if required.

(4) Transfer the whole blood to an appropriately sized sterile container (e.g., sterile flask or centrifuge tube). If the blood was drawn into multiple containers (e.g., multiple blood collection tubes), pool the blood in one sterile container.

Note: While transferring the blood, determine the exact blood volume.

(5) Dilute the blood with PBS in a 1:2.5 ratio and mix gently.

Example: Add 15 ml PBS to 10 ml whole blood.

(6) Aspirate the blood/PBS mixture in a serological pipette and transfer 25–30 ml per tube to SepMate™-50 tubes filled with Lymphoprep™.
  (a) Point the tip of the serological pipette filled with blood/PBS to the inner wall of the SepMate™-50 tube.
  (b) Slowly and carefully pipette the blood/PBS onto the inner wall of the tube.

Note: Avoid pipetting through the central hole to avoid mixing of the blood/PBS with the Lymphoprep™ below the insert. If you observe strong turbulences below the insert, stop pipetting, shift the position of the serological pipette at the inner wall of the tube further to the left or right, and slowly continue pipetting.

(7) Close the SepMate™-50 tubes filled with blood/PBS and centrifuge 10 min at 1,200 ×g at RT. The break of the centrifuge may be left on for this procedure.

Note: After the centrifugation the PBMCs will have accumulated in the plasma as a whitish-grey layer above the insert. Density gradient medium, red blood cells, and granulocytes are located below the insert.

(8) Pour the plasma containing the PBMCs to fresh sterile containers with a swift motion. Pool the plasma with cells of one blood donor where necessary.

Attention: Do not hold the SepMate™-50 tubes in an upside-down position for longer than 2 s! Otherwise, red blood cells and density gradient medium can flow past the insert and contaminate the PBMCs.

#### MEK1/2 inhibitor treatment

If the blood was derived from a clinical trial where probands were treated with MEK1/2 inhibitors, this section can be skipped. In this case, continue immediately with the PMA stimulation.

(1) Resuspend the PBMCs in the plasma and divide into the desired number of groups in separate 50 ml centrifuge tubes.

Example: six groups: baseline control, positive control, solvent control, and three groups treated with different concentrations of the MEK1/2 inhibitor.

Note: Always prepare a baseline (no treatment, no stimulation), positive (no treatment, PMA stimulation), and solvent control (treatment with the solvent in which the MEK1/2 inhibitor is prepared, PMA stimulation), to assess minimal and maximal MEK1/2 activation, as well as any solvent effects.

(2) Prepare 10 µl MEK1/2 inhibitor working solution (WS) in PBS per 1 ml PBMC suspension. The concentration of the WS needs to be 100-times higher than the final concentration desired for cell treatment.

Example: The desired treatment concentration with the MEK1/2 inhibitor is 10 μg/ml in 10 ml cell suspension, therefore prepare at least 100 µl of a 1 mg/ml WS in PBS.

Note: Prepare a solvent WS with the same solvent concentration as present in the MEK1/2 inhibitor WS.

Attention: In case of DMSO used as a solvent, do not expose the cells to concentrations exceeding 1% DMSO to avoid cytotoxic effects. Keep in mind that the subsequent stimulation with PMA is carried out in a final DMSO concentration of 0.1%.

(3) Add the MEK1/2 inhibitor and solvent WS, respectively, to the PBMCs suspended in plasma in a 1:100 dilution. Mix gently by pipetting up and down 3 times and gently inverting the tubes two times.

Example: Add 100 µl WS to PBMCs suspended in 10 ml plasma.

Attention: Do not add anything to the baseline and positive control but mix gently.

(4) Incubate the cells at 37°C for 1 h preventing from sedimentation by gently inverting the tubes two times every 10–15 min.

#### PMA stimulation

The PBMCs will be stimulated with a final concentration of 400 nM PMA in 0.1% DMSO.

(1) Prepare 10 µl of a 40 µM PMA WS in 10% DMSO per 1 ml PBMC suspension.

Note: The concentration of the PMA WS needs to be 100-times higher than the final concentration intended for cell stimulation.

Example: Prepare at least 100 µl 40 µM PMA WS for 10 ml PBMC suspension. Start from a 400 µM PMA stock solution prepared in 100% DMSO. Dilute the 400 µM PMA stock solution 1:10 in PBS to obtain the 40 µM PMA WS in 10% DMSO. Thoroughly mix by vortexing 5 s.

(2) Carefully resuspend the PBMCs in the plasma.
(3) Add the PMA WS to the PBMCs suspended in plasma in a 1:100 dilution. Mix gently by pipetting up and down 3 times and gently inverting the tubes two times.

Example: Add 100 µl WS to PBMCs suspended in 10 ml plasma.

Attention: Do not add anything to the baseline control but mix gently as described before.

(4) Incubate the cells at 37°C for 30 min preventing from sedimentation by gently inverting the tubes two times every 10–15 min.

#### PBMC purification

(1) Fill each tube containing PBMCs up to 50 ml with PBS.
  (a) Close the tubes and mix by gently inverting 3 times.
(2) Centrifuge 8 min at 300 ×g at RT.
  (a) Decant and discard the supernatant in one swift motion.
  (b) Close the tubes and resuspend the pellets (e.g., by scraping the tubes 3 times across the holes for air circulation in the cell culture bench).
  (c) Fill the tubes up to 30 ml with PBS.
  (d) Close the tubes and mix by gently inverting 3 times.

Attention: At step b, the pellet might appear translucent. This does not affect the outcome of the results. Proceed as with a visible pellet.

(3) Centrifuge 8 min at 300 ×g at RT.
  Attention: Perform the procedure (a–b) described in the following with one tube at a time.
  (a) Decant and discard the supernatant in one swift motion and keep the tube upside-down to avoid disturbing the cell pellet by backflow of supernatant.
  (b) Remove the remaining supernatant on the pellet or clinging as droplets to the wall of the tube using a 200 µl pipette. Go as close to the pellet as possible without removing any cells from the pellet. Close the tube.

Note: Approx. 100 µl supernatant will still remaining with the cell pellet after this procedure. This will not affect the outcome of the results.

Attention: In case of a translucent pellet (which will not affect the outcome of the results) handle especially careful at step b. You need to keep track of the assumed position of the (invisible) pellet to not aspirate and lose the pellet during removal of the supernatant.

(4) Continue with your method of choice for cell lysis and biomarker analysis for the ERK1/2 phosphorylation status.

Note: Although assessment of the ERK1/2 phosphorylation status using the Wes™ technology and the Meso Scale Discovery system is described in detail in this manuscript, other methods of choice (such as Western blotting or flow cytometry analysis using phenotypic markers) to assess ERK1/2 activation levels can be used after the PBMC isolation. Note that this manuscript does not include a protocol for ERK1/2 phosphorylation status assessment using other methods, and that these methods have to be established and optimized by the user.

## Results

### Strong MEK1/2 activation in PBMCs after stimulation with PMA

**FIGURE 2:**
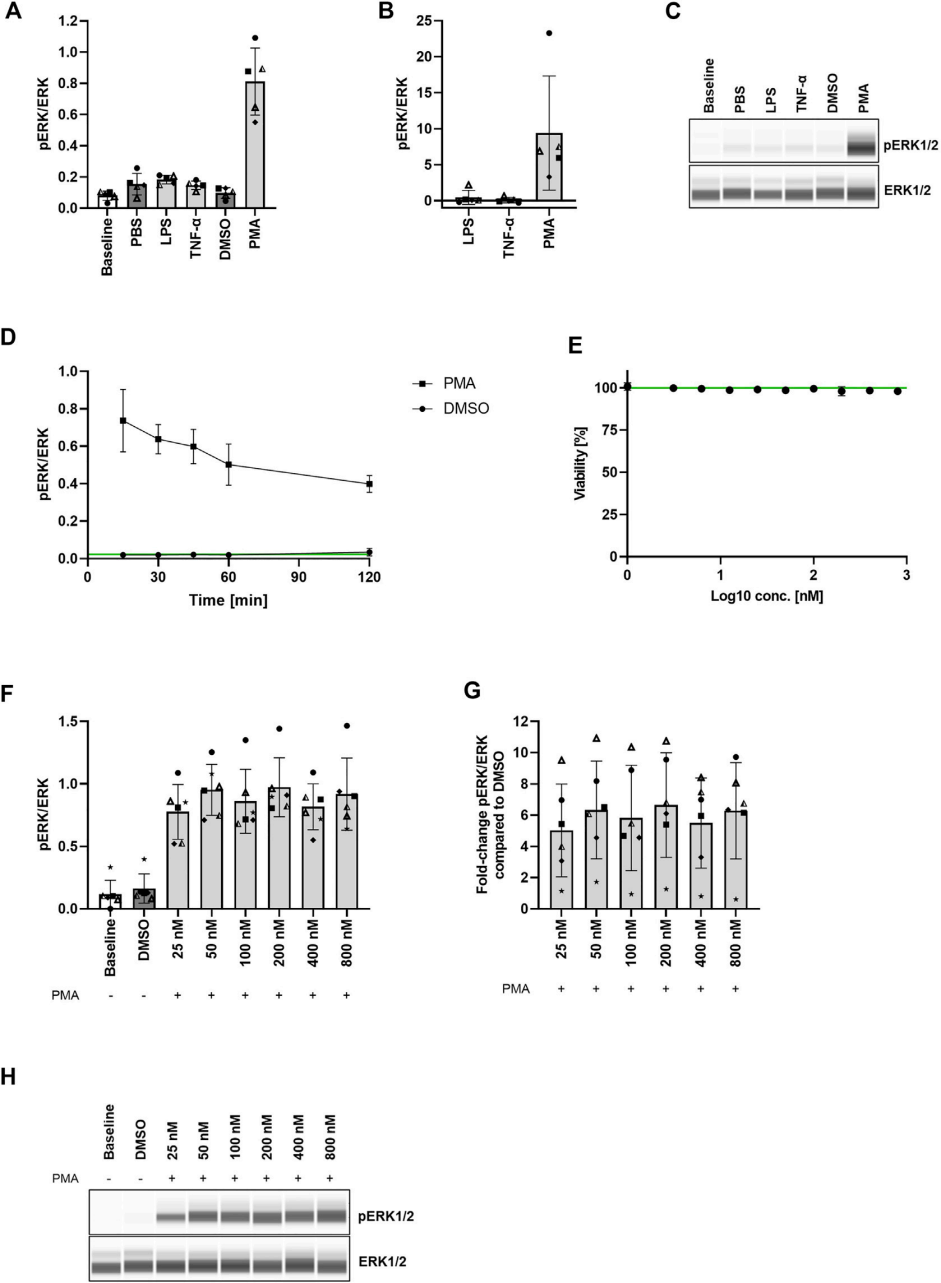
Strong MEK1/2 activation without cytotoxic effects in PBMCs after 30 min stimulation with PMA. **(A,B)** Comparison of ERK1/2 phosphorylation levels in PBMCs after stimulation with LPS, TNF-α, and PMA. PBMCs were stimulated with 1 μg/ml LPS, 20 ng/ml TNF-α, or 400 nM PMA. The cells were purified, lysed, and the ERK1/2 phosphorylation status was assessed with the Wes™ system. Data are presented as individual values and mean with standard deviation. Each datapoint represents one blood donor. *N* = 5 blood donors. **(C)** Presentation of pERK1/2 and ERK1/2 levels as Western blot-like bands of one representative blood donor included in **(A,B)**. The Western blot-like band data of all blood donors is shown in the Supplementary Material. **(D)** PMA stimulation timeline. PBMCs were stimulated with 400 nM PMA for 15–120 min, the cells were purified, lysed, and the ERK1/2 phosphorylation status was assessed with the Wes™ system. Data are presented as mean with standard deviation. *N* = 3 blood donors. **(E)** PMA cytotoxicity assessment. PBMCs were treated with 3.13–800 nM PMA for 45 min. Cytotoxicity was assessed with LDH release assay. Data are presented as mean with standard deviation. *N* = 3 blood donors with *n* = 3 technical replicates each. **(F,G)** PMA titration. PBMCs were stimulated with 25–800 nM PMA for 30 min and the ERK1/2 phosphorylation status was assessed with the Wes™ system. Data are presented as individual values and mean with standard deviation. Each datapoint represents one blood donor. *N* = 6 blood donors. **(H)** Presentation of pERK1/2 and ERK1/2 levels as Western blot-like bands of one representative blood donor included in **(F,G)**. The Western blot-like band data of all blood donors is shown in the Supplementary Material.

First, we wanted to assess the stimulus required for the highest possible MEK1/2 activation in human PBMCs in absence of any cytotoxic effects. Therefore, we isolated PBMCs from whole blood of healthy volunteers and treated the cells with 1 μg/ml LPS, 20 ng/ml TNF-α, or 400 nM PMA for 30 min. Since ERK1/2 is the only downstream target of MEK1/2, we assessed the levels of ERK1/2 phosphorylation with the Wes™ system as a measure for MEK1/2 activation. After treatment with LPS and TNF-α we observed only negligible changes in the ERK1/2 phosphorylation levels compared to the control. The mean ratio of phosphorylated ERK1/2 to total ERK1/2 (in the following referred to as pERK/ERK) after LPS treatment (0.18 ± 0.03) was 0.47-fold higher (range: −0.18 to 2.20-fold) than the pERK/ERK ratio measured in the control (0.15 ± 0.07). Treatment with TNF-α increased the mean pERK/ERK ratio (0.15 ± 0.03) 0.07-fold (range: −0.29 to 0.66-fold) compared to the pERK/ERK ratio in the control (0.15 ± 0.07). In contrast, treatment with PMA resulted in the highest increase of pERK/ERK (0.81 ± 0.22) of 9.38-fold (range: 3.30 to 23.28-fold) compared to the control (0.10 ± 0.03) (Figures 2A–C; Supplementary Figure S1). As changes of the MEK1/2 activation status upon MEK1/2 inhibitor treatment can be identified more easily in a larger range between baseline and maximal MEK1/2 activation (no MEK1/2 inhibition), we considered a > 2-fold elevation from baseline MEK1/2 activation (and therefore stimulation with PMA) as suitable for MEK1/2 inhibition experiments. MEK1/2 activation < 2-fold from baseline may prove unreliable and therefore needs to be considered with care.

Next, we assessed the optimal duration of PMA stimulation in PBMCs, which would achieve the highest MEK1/2 activation. Therefore, we treated PBMCs with 400 nM PMA in different intervals from 15 to 120 min and determined the pERK/ERK ratio (ratio of phosphorylated ERK1/2 to total ERK1/2) as a measure of MEK1/2 activation. The pERK/ERK ratio measured after 0 min (no PMA treatment) indicated a baseline level of 0.02 ± 0.002. The mean pERK/ERK ratio in the DMSO control remained at baseline level throughout the whole treatment time (0.02 ± 0.01). 15 min PMA treatment resulted in a pERK/ERK ratio of 0.74 ± 0.17. The ratio decreased to 0.64 ± 0.08 after 30 min, and decreased further to 0.60 ± 0.09, 0.50 ± 0.11, and 0.40 ± 0.05 after 45, 60, and 120 min, respectively (Figure 2D). Although the highest level of phosphorylated ERK1/2 was measured after 15 min treatment, we decided to treat the cells for 30 min with PMA in all subsequent experiments, since 30 min treatment resulted in only a slightly lower ERK1/2 phosphorylation but with a much higher consistency between the blood donors than 15 min treatment.

To determine the effect of PMA treatment on cell viability, we treated PBMCs with 3.13–800 nM PMA for 45 min and assessed the cell viability *via* LDH release. We demonstrated that none of the applied concentrations mediated cytotoxic effects within 45 min treatment (Figure 2E).

Lastly, to assess the PMA concentration which yields the highest ERK1/2 phosphorylation compared to the DMSO control, we stimulated PBMCs with 25–800 nM PMA for 30 min. The pERK/ERK ratio (ratio of phosphorylated ERK1/2 to total ERK1/2) of the DMSO control (0.16 ± 0.12) roughly resembled the baseline pERK/ERK ratio (0.12 ± 0.11). Treatment with all PMA concentrations induced comparable MEK1/2 activation levels reaching from a mean fold-increase of the pERK/ERK ratio compared to the DMSO control of 5.02 ± 2.97 to a mean fold-increase of 6.65 ± 3.35 (Figures 2F–H; Supplementary Figure S2). These data clearly indicate that a MEK1/2 activation plateau has been reached upon treatment with all applied concentrations. Therefore, we decided to use 400 nM PMA for stimulation in all subsequent experiments, since this concentration was successfully used before for ERK1/2 phosphorylation induction in PBMCs (Yoon & Seger, 2006).

In some cases, we observed the pitfall that after stimulation with PMA the PBMC pellet appeared translucent during PBMC purification. Since this has no effect on the outcome of the results, we recommend continuing the procedure as with a visible pellet. Here, it is especially important to keep track of the assumed position of the (translucent) pellet in the tube to avoid losing the pellet at some step.

### Zapnometinib treatment inhibits MEK1/2 in PBMCs

**FIGURE 3:**
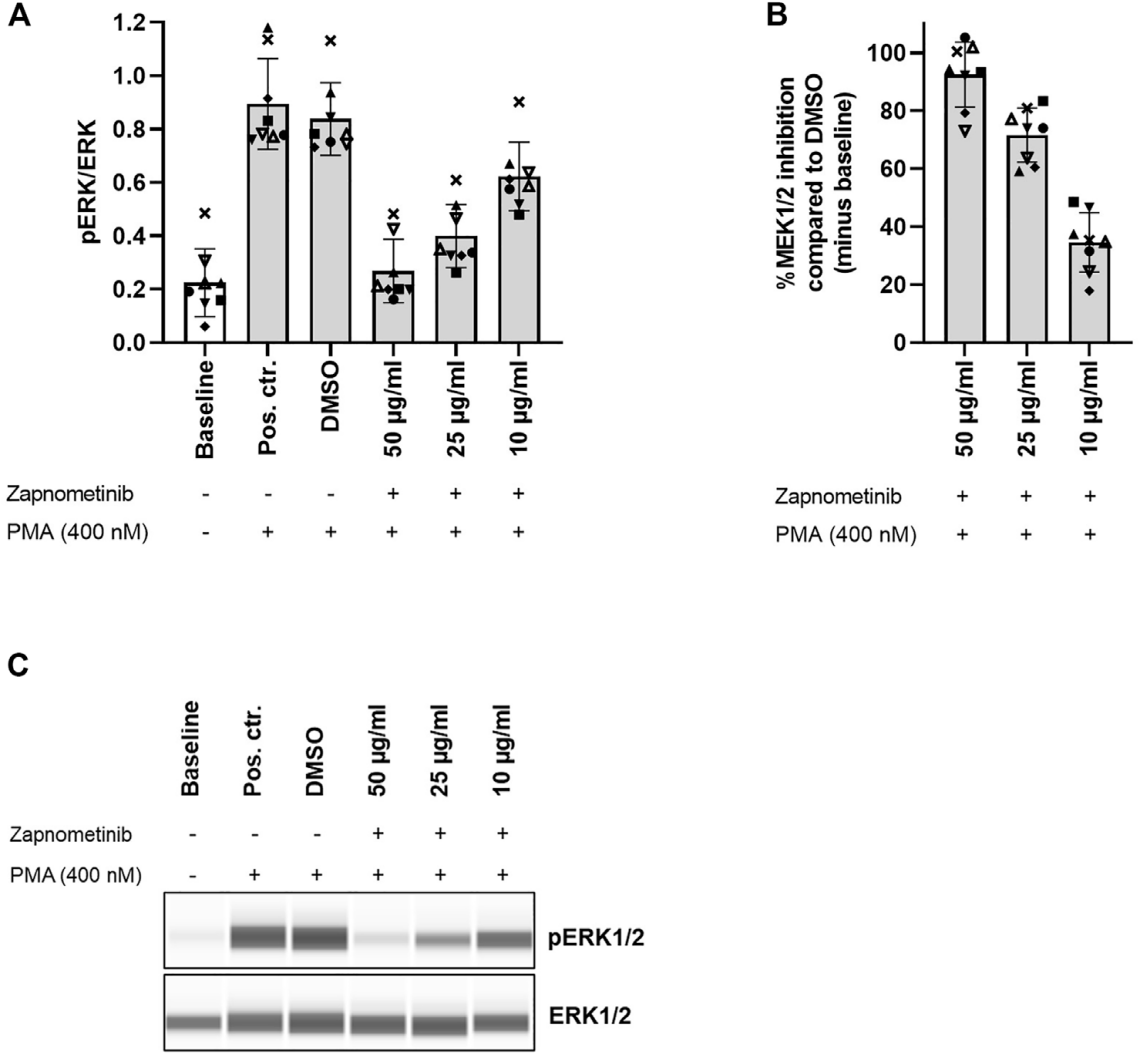
Zapnometinib inhibits MEK1/2 in human PBMCs. PBMCs were treated with 10, 25, and 50 μg/ml zapnometinib, followed by stimulation with PMA. The ERK1/2 phosphorylation status was assessed with the Wes™ system. Data are displayed as **(A)** ratio pERK/ERK (ratio of phosphorylated ERK1/2 to total ERK1/2) and **(B)** %MEK1/2 inhibition compared to DMSO. Data are presented as individual values and mean with standard deviation. Each datapoint represents one blood donor. *N* = 8 blood donors. **(C)** Presentation of pERK1/2 and ERK1/2 levels as Western blot-like bands of one representative blood donor included in **(A,B)**. The Western blot-like band data of all blood donors is shown in the Supplementary Material.

To assess zapnometinib-induced MEK1/2 inhibition in human PBMCs, we treated PBMCs with 10, 25, and 50 μg/ml zapnometinib and subsequently stimulated with 400 nM PMA. We determined the ratio of phosphorylated ERK1/2 to total ERK1/2 (pERK/ERK) as a measure for MEK1/2 activation. The baseline pERK/ERK ratio measured without treatment or stimulation was 0.22 ± 0.13, which increased to 0.89 ± 0.17 or 0.84 ± 0.14 upon PMA stimulation without previous treatment (positive control or DMSO control, respectively). The pERK/ERK ratio in the DMSO control (PMA stimulation after DMSO treatment) was defined as 100% MEK1/2 activation. We found that treatment with 10 or 25 μg/ml zapnometinib induced MEK1/2 inhibition of 34.6 ± 10.2% or 71.6 ± 9.3%, respectively. Treatment with 50 μg/ml zapnometinib inhibited MEK1/2 by 92.4 ± 11.2% and therefore maintained the MEK1/2 activation roughly at baseline level (Figure 3; Supplementary Figure S3).

These data demonstrate that, without MEK1/2 inhibitor treatment, MEK1/2 stimulation with PMA induces high MEK1/2 activation, which is clearly distinguishable from baseline MEK1/2 activation in human PBMCs. Simultaneously, treatment with zapnometinib can maintain MEK1/2 activation levels close to baseline despite subsequent stimulation with PMA.

This constitutes the main advantage of our method since it allows for assessment of MEK1/2 inhibition compared to a control with high MEK1/2 activation instead of comparison to a baseline control with already low MEK1/2 activation.

### Comparable results for cell lysis using 1x RIPA or Complete lysis buffer

We further aimed to investigate the reproducibility of our data when assessed with different systems. We therefore analyzed the same samples with the Wes™ and the MSD platform in parallel. However, both systems use different methods for sample preparation. Hence, as a first step, we investigated whether the Wes™ system could process samples obtained by cell lysis with Complete lysis buffer (used by the MSD platform) and whether this would yield comparable readouts to samples obtained by cell lysis with 1x RIPA buffer (used by the Wes™ system).

**FIGURE 4:**
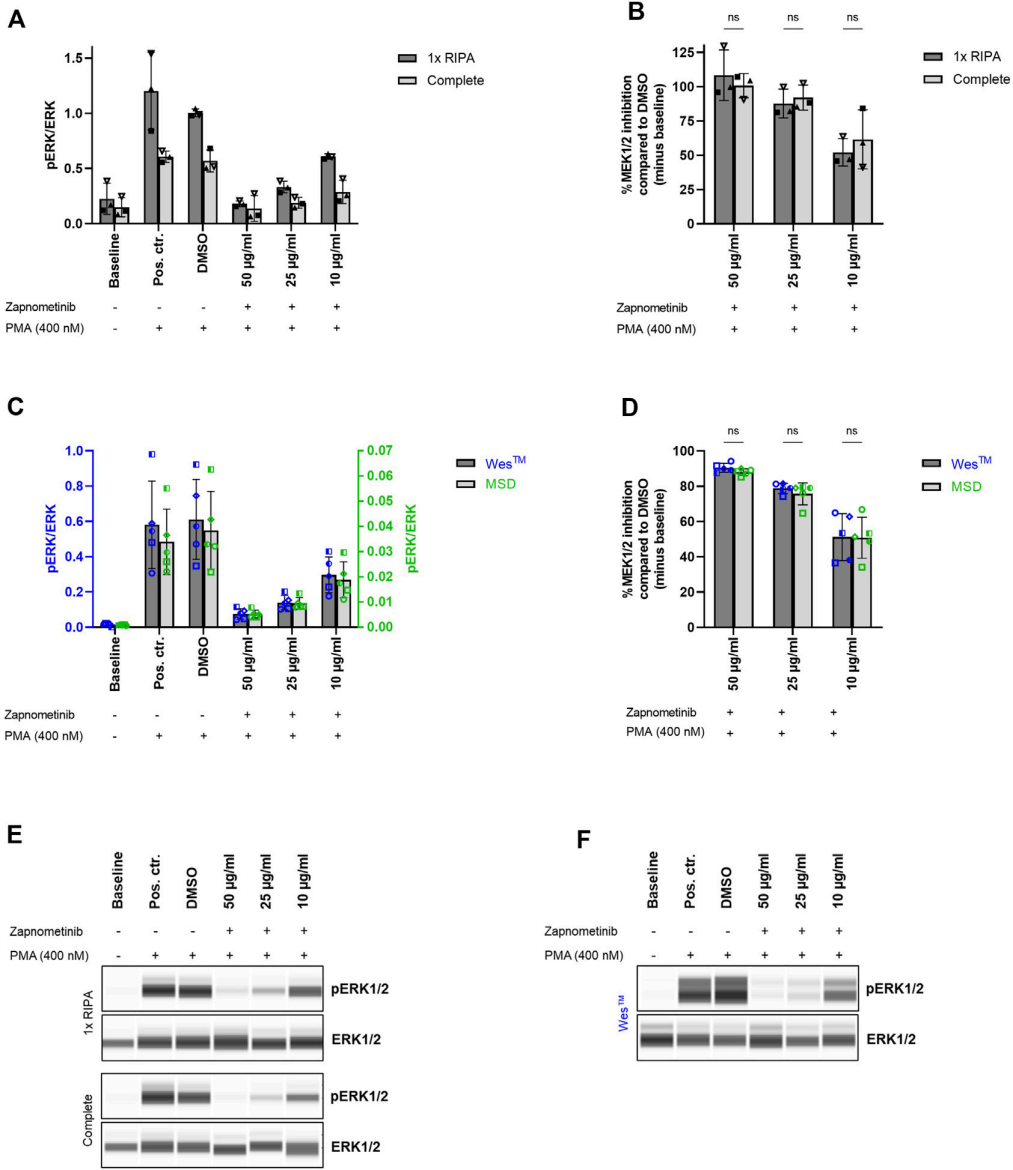
Comparable results for sample analysis with Wes™ and MSD system. **(A,B)** Comparison of cell lysis using 1x RIPA or Complete lysis buffer as preliminary step for Wes™ and MSD system comparison. Cell treatment was performed as previously described and cells were lysed using either 1x RIPA (dark grey) or Complete lysis buffer (light grey). The ERK1/2 phosphorylation status was assessed with the Wes™ system. Two-way ANOVA with Šídák correction was used for statistical analysis to compare the results obtained for cell lysis using 1x RIPA or Complete lysis buffer. ns, not statistically significant. *N* = 3 blood donors. **(C,D)** Comparison of **(C)** pERK/ERK (ratio of phosphorylated ERK1/2 to total ERK1/2) and **(D)** %MEK1/2 inhibition upon sample analysis with the Wes™ and the MSD system. Cell treatment and lysis was performed as previously described and the samples were analyzed with the Wes™ (dark grey bars, blue datapoints) and the MSD system (light grey bars, green datapoints). Two-way ANOVA with Šídák correction was used for statistical analysis to compare the results obtained with Wes™ and MSD platform. ns, not statistically significant. *N* = 5 blood donors. Data are displayed as **(A,C)** ratio pERK/ERK and **(B,D)** %MEK1/2 inhibition compared to DMSO. Data are presented as individual values and mean with standard deviation. Each datapoint represents one blood donor. **(E,F)** Presentation of pERK1/2 and ERK1/2 levels as Western blot-like bands of one representative blood donor included in **(A,B)** or **(C,D)**, respectively. The Western blot-like band data of all blood donors is shown in the Supplementary Material.

Therefore, PBMCs were treated with 10, 25, and 50 μg/ml zapnometinib and subsequently stimulated with 400 nM PMA. Each sample was divided into two equal parts, of which one was lysed using either 1x RIPA or Complete lysis buffer. The pERK/ERK ratio (ratio of phosphorylated ERK1/2 to total ERK1/2) was determined with the Wes™ system as a measure for MEK1/2 activation. The baseline pERK/ERK ratio measured after cell lysis with 1x RIPA buffer was 0.23 ± 0.14 and was therefore higher than the baseline pERK/ERK ratio of 0.15 ± 0.09 measured after lysis with Complete lysis buffer. A comparable pattern could be observed for all conditions. The pERK/ERK ratio of the positive or DMSO control was 1.20 ± 0.35 and 0.61 ± 0.05, or 1.00 ± 0.04 and 0.57 ± 0.10 after cell lysis using 1x RIPA and Complete lysis buffer, respectively. Upon treatment with 10, 25, or 50 μg/ml zapnometinib, we measured a pERK/ERK ratio of 0.61 ± 0.02 and 0.29 ± 0.11, 0.33 ± 0.05 and 0.19 ± 0.05, or 0.18 ± 0.03 and 0.14 ± 0.12 after cell lysis with 1x RIPA and Complete lysis buffer (Figures 4A,E; Supplementary Figure S4).

Although the cell lysis method affected the measured pERK/ERK ratios, the MEK1/2 inhibition compared to the DMSO control remained unaffected by the method of cell lysis. MEK1/2 inhibition by 52.1 ± 10.0% and 61.6 ± 21.6%, 87.7 ± 10.5% and 92.1 ± 9.1%, or 108.4 ± 18.4% and 100.8 ± 8.8% was measured upon treatment with 10, 25, or 50 μg/ml zapnometinib, and cell lysis with 1x RIPA and Complete lysis buffer, respectively. Two-way ANOVA with Šídák correction was used for statistical comparison of the results obtained after cell lysis with 1x RIPA or Complete lysis buffer. The test revealed no statistically significant differences (Figure 4B).

These data demonstrate that both 1x RIPA and Complete lysis buffer can be used for cell lysis when the lysates are to be analyzed with the Wes™ system.

### Comparable results for sample analysis with Wes™ and Meso Scale Discovery systems

As a next step, we aimed to compare ERK1/2 phosphorylation measured either by the Wes™ or the MSD system. Therefore, PBMCs were treated with 10, 25, and 50 μg/ml zapnometinib and subsequently stimulated with 400 nM PMA. Cell lysates were prepared using Complete lysis buffer and were analyzed with the Wes™ and the MSD system for their ERK1/2 phosphorylation status. The pERK/ERK ratio (ratio of phosphorylated ERK1/2 to total ERK1/2) was then used as a measure for MEK1/2 activation.

Using the Wes™ system, we detected a baseline pERK/ERK ratio of 0.02 ± 0.01, which increased to 0.58 ± 0.25 or 0.61 ± 0.23 in the positive or the DMSO control. Treatment with 10, 25, and 50 μg/ml zapnometinib reduced the pERK/ERK ratio to 0.30 ± 0.10, 0.14 ± 0.04, and 0.08 ± 0.03 (Figures 4C,F; Supplementary Figure S5). With the MSD system we detected pERK/ERK levels which were overall more than 10-times lower than the pERK/ERK ratios detected with the Wes™ system. Using the MSD platform, a baseline pERK/ERK ratio of 0.001 ± 0.0002 was detected. The ratio increased to 0.03 ± 0.01 or 0.04 ± 0.02 in the positive or DMSO control. Zapnometinib treatment with 10, 25, and 50 μg/ml reduced the ratio to 0.02 ± 0.01, 0.01 ± 0.002, and 0.005 ± 0.002 (Figure 4C).

However, when compared to the DMSO control, the pERK/ERK ratios detected with both platforms resulted in almost identical %MEK1/2 inhibition values. With the Wes™ system we detected 51.2 ± 13.3%, 78.8 ± 2.9%, or 90.5 ± 2.5% MEK1/2 inhibition after treatment with 10, 25, or 50 μg/ml zapnometinib. In comparison, using the same treatment, we measured 50.9 ± 11.7%, 75.5 ± 6.2%, or 88.0 ± 1.9% MEK1/2 inhibition using the MSD platform. Two-way ANOVA with Šídák correction was used for statistical comparison of the results obtained with the Wes™ and MSD platform. The test revealed no statistically significant differences (Figure 4D).

This highlights that referencing to a control is essential to obtain data comparability across different systems, instead of focusing on pERK/ERK ratios alone. Furthermore, these data demonstrate that both systems are of high accuracy and yield highly reproducible results. In addition, this emphasizes the robustness of the method we developed, since almost identical results were obtained with two different systems used for biomarker analysis.

## Discussion

Assessing target engagement of MEK1/2 inhibitors in clinical trials with healthy participants for drug development can be challenging due to the low basal MEK1/2 activation. Therefore, robust methods to easily assess MEK1/2 inhibitor target engagement are urgently needed. We hence developed a method that will use PBMCs isolated from whole blood of MEK1/2 inhibitor-treated study participants, followed by *ex vivo* stimulation with PMA to activate MEK1/2. Impaired MEK1/2 activation after PMA stimulation is a consequence of MEK1/2 inhibition after MEK1/2 inhibitor treatment. Although our method was not yet applied *in vivo*, following our approach in *in vitro* proof of principle experiments, we showed that, without MEK1/2 inhibitor treatment, MEK1/2 stimulation with PMA induces high MEK1/2 activation. As changes of the MEK1/2 activation status following MEK1/2 inhibitor treatment can be identified more easily in a larger range between baseline and maximal MEK1/2 activation (no MEK1/2 inhibition), we considered a > 2-fold increase above baseline MEK1/2 activation as suitable for MEK1/2 inhibition experiments. Simultaneously, we demonstrated that treatment with the MEK1/2 inhibitor zapnometinib can inhibit PMA-mediated MEK1/2 activation to reach approximately baseline level. We observed comparable results in datasets where uninhibited MEK1/2 was activated < 2-fold compared to baseline. This demonstrates the robustness of the developed method. However, as MEK1/2 activation < 2-fold from baseline may result unreliable, the significance of the results may be compromised. We hence recommend considering activation of uninhibited MEK1/2 < 2-fold from baseline with care. As these findings were reproducible across different analytic systems, the robustness of our method is further supported.

Several researchers used methods to assess MEK1/2 activation/inhibition in human PBMCs, which feature aspects that are comparable to our method. However, all these methods have limitations that our approach can overcome. LoRusso et al. (2005) adapted a method from Sebolt-Leopold et al. (2003). The team sampled blood from MEK1/2 inhibitor-treated cancer patients, stimulated the whole blood with PMA, and assessed the MEK1/2 inhibition in isolated PBMCs. In 2009, Lee et al. determined the PD of a MEK1/2 inhibitor intended for cancer treatment in healthy volunteers. Similar to LoRusso et al. (2005), they sampled blood from treated subjects and stimulated the whole blood with PMA to activate MEK1/2 in PBMCs (Lee et al., 2009). When we repeated the PBMC stimulation in whole blood, we noticed that some erythrocytes had undergone hemolysis (indicated by hemolytic plasma) and that some red blood matter had accumulated within the PBMC layer after PBMC isolation using SepMate™-50 tubes. We therefore introduced an additional erythrocyte lysis step before proceeding. Therefore, we see a major disadvantage of stimulating PBMCs in whole blood, since it requires an additional red blood cell lysis step, which makes the protocol more labor-intensive and prone to handling errors. This disadvantage was overcome by Jamieson et al. (2016). In their study, cancer patients were treated with a MEK1/2 inhibitor and whole blood was sampled at different timepoints. PBMCs were isolated using cell preparation tube (CPT™) vacutainers. The cells were kept in autologous plasma obtained during isolation and were stimulated with PMA. Therefore, no red blood cell lysis was required. However, one major limitation we found with this protocol is the PBMC incubation in the “concentrated” plasma obtained during the isolation. Whole blood contains approximately 55% plasma (Frick, 2003). In theory, upon PBMC isolation following the method introduced by Jamieson et al. (2016), PBMCs and plasma-bound substances (such as MEK1/2 inhibitors with high plasma binding capacity) are concentrated in ca. 55% of their initial volume. For instance, given 1 ml whole blood contains 1 µg of a compound, this equals a concentration of 1 μg/ml. Assuming this compound features high plasma binding properties, plasma separation from whole blood results in 1 µg compound being concentrated in 550 µl plasma, which eventually amounts to a concentration of 1.8 μg/ml. Maintaining the isolated PBMCs in this plasma, therefore exposes the cells to 1.8-times the concentration of the compound that was initially present *in vivo*. By experiment, we observed an even stronger concentration effect of 2.5-times (data not shown). Our method addresses this issue by diluting the whole blood for PBMC isolation in PBS and maintaining the PBMCs in “diluted” plasma to maintain *in vivo*-like conditions.

Taken together, one advantage of our method is the user-friendly and easy-to-follow protocol with as few steps as possible. However, the major advantage of our approach over previous ones is the maintenance of *in vivo*-like conditions by substitution of the red blood cell fraction with PBS during PBMC isolation.

The optimization of the protocol to enable performing the PMA stimulation in plasma for easier handling, however, also leads to limitations of our method. This method is only applicable when the MEK1/2 inhibitor, which was initially present in the whole blood, can be extracted with the plasma and the cells during the PBMC isolation. Otherwise, the inhibitor is removed from the cells prior to PMA stimulation. If the inhibitor does not feature a prolonged residence on MEK1/2, the PMA stimulation in absence of the inhibitor might eventually distort the results. In this case, PMA stimulation in whole blood is unavoidable and an additional erythrocyte lysis step after PBMC isolation is required. This step makes the procedure slightly more complex but can be easily included in our protocol. Another limitation of the study is that at present we cannot conclude on the influence of upscaling on the quality of the results. If results from PD studies are correlated with pharmacological results, this can lead to an increased sample throughput in the laboratory. Therefore, we would recommend not to perform PD analyses at all time points at which pharmacokinetic (PK) analyses are performed, but to focus on selected time points.

In conclusion, we developed a novel method that can be suitable for the assessment of MEK1/2 inhibition in PBMCs in clinical trials. Our protocol does not require expensive laboratory equipment, it is easy to follow, and maintains the cells in an *in vivo*-like condition throughout the whole handling process. Based on the robust and reproducible results we obtained following our method in *in vitro* experiments, we consider this approach a promising advance for the easy assessment of MEK1/2 inhibitor target engagement in healthy clinical trials subjects.

## Data Availability

The original contributions presented in the study are included in the article/Supplementary Material, further inquiries can be directed to the corresponding author.

## References

[B1] Ciuffreda L. Del Bufalo D. Desideri M. Di Sanza C. Stoppacciaro A. Ricciardi M. R. (2009). Growth-inhibitory and antiangiogenic activity of the MEK inhibitor PD0325901 in malignant melanoma with or without BRAF mutations. Neoplasia 11 (8), 720–731. 10.1593/neo.09398 19649202 PMC2713590

[B2] Frick P. (2003). Blut- und Knochenmarksmorphologie. New York, United States: Georg Thieme Verlag. 10.1055/b-002-44929

[B3] Infante J. R. Fecher L. A. Falchook G. S. Nallapareddy S. Gordon M. S. Becerra C. (2012). Safety, pharmacokinetic, pharmacodynamic, and efficacy data for the oral MEK inhibitor trametinib: A phase 1 dose-escalation trial. Lancet. Oncol. 13 (8), 773–781. 10.1016/S1470-2045(12)70270-X 22805291

[B4] Iverson C. Larson G. Lai C. Yeh L. T. Dadson C. Weingarten P. (2009). RDEA119/BAY 869766: A potent, selective, allosteric inhibitor of MEK1/2 for the treatment of cancer.Sep 1). RDEA119/BAY 869766: A potent, selective, allosteric inhibitor of MEK1/2 for the treatment of cancer. Cancer Res. 69 (17), 6839–6847. 10.1158/0008-5472.CAN-09-0679 19706763

[B5] Jamieson D. Griffin M. J. Sludden J. Drew Y. Cresti N. Swales K. (2016). A phase I pharmacokinetic and pharmacodynamic study of the oral mitogen-activated protein kinase kinase (MEK) inhibitor, WX-554, in patients with advanced solid tumours. Eur. J. Cancer 68, 1–10. 10.1016/j.ejca.2016.08.026 27693888

[B6] Koch-Heier J. Schonsiegel A. Waidele L. M. Volk J. Full Y. Wallasch C. (2022). Pharmacokinetics, pharmacodynamics and antiviral efficacy of the MEK inhibitor zapnometinib in animal models and in humans. Front. Pharmacol. 13, 893635. 10.3389/fphar.2022.893635 35784712 PMC9240354

[B7] Laure M. Hamza H. Koch-Heier J. Quernheim M. Muller C. Schreiber A. (2020). Antiviral efficacy against influenza virus and pharmacokinetic analysis of a novel MEK-inhibitor, ATR-002, in cell culture and in the mouse model. Antivir. Res. 178, 104806. 10.1016/j.antiviral.2020.104806 32304723

[B8] Lee L. Niu H. Rueger R. Igawa Y. Deutsch J. Ishii N. (2009). The safety, tolerability, pharmacokinetics, and pharmacodynamics of single oral doses of CH4987655 in healthy volunteers: Target suppression using a biomarker. Clin. Cancer Res. 15 (23), 7368–7374. 10.1158/1078-0432.CCR-09-1696 19934286

[B9] Li D. March M. E. Gutierrez-Uzquiza A. Kao C. Seiler C. Pinto E. (2019). ARAF recurrent mutation causes central conducting lymphatic anomaly treatable with a MEK inhibitor. Nat. Med. 25 (7), 1116–1122. 10.1038/s41591-019-0479-2 31263281

[B10] Lorusso P. M. Adjei A. A. Varterasian M. Gadgeel S. Reid J. Mitchell D. Y. (2005). Phase I and pharmacodynamic study of the oral MEK inhibitor CI-1040 in patients with advanced malignancies. J. Clin. Oncol. 23 (23), 5281–5293. 10.1200/JCO.2005.14.415 16009947

[B11] Ludwig S. Planz O. Pleschka S. Wolff T. (2003). Influenza-virus-induced signaling cascades: Targets for antiviral therapy? Trends Mol. Med. 9 (2), 46–52. 10.1016/s1471-4914(02)00010-2 12615037

[B12] McDaid H. M. Lopez-Barcons L. Grossman A. Lia M. Keller S. Perez-Soler R. (2005). Enhancement of the therapeutic efficacy of taxol by the mitogen-activated protein kinase kinase inhibitor CI-1040 in nude mice bearing human heterotransplants. Cancer Res. 65 (7), 2854–2860. 10.1158/0008-5472.CAN-04-4391 15805287

[B13] Meier F. Schittek B. Busch S. Garbe C. Smalley K. Satyamoorthy K. (2005). The RAS/RAF/MEK/ERK and PI3K/AKT signaling pathways present molecular targets for the effective treatment of advanced melanoma. Front. Biosci. 10, 2986–3001. 10.2741/1755 15970553

[B14] Planz O. (2013). Development of cellular signaling pathway inhibitors as new antivirals against influenza. Antivir. Res. 98 (3), 457–468. 10.1016/j.antiviral.2013.04.008 23603495

[B15] Pleschka S. Wolff T. Ehrhardt C. Hobom G. Planz O. Rapp U. R. (2001). Influenza virus propagation is impaired by inhibition of the Raf/MEK/ERK signalling cascade. Nat. Cell Biol. 3 (3), 301–305. 10.1038/35060098 11231581

[B16] Schreiber A. Viemann D. Schoning J. Schloer S. Mecate Zambrano A. Brunotte L. (2022). The MEK1/2-inhibitor ATR-002 efficiently blocks SARS-CoV-2 propagation and alleviates pro-inflammatory cytokine/chemokine responses. Cell. Mol. Life Sci. 79 (1), 65. 10.1007/s00018-021-04085-1 35013790 PMC8747446

[B17] Sebolt-Leopold J. S. Dudley D. T. Herrera R. Van Becelaere K. Wiland A. Gowan R. C. (1999). Blockade of the MAP kinase pathway suppresses growth of colon tumors *in vivo* . Nat. Med. 5 (7), 810–816. 10.1038/10533 10395327

[B18] Sebolt-Leopold J. S. (2004). MEK inhibitors: A therapeutic approach to targeting the ras-MAP kinase pathway in tumors. Curr. Pharm. Des. 10 (16), 1907–1914. 10.2174/1381612043384439 15180527

[B19] Sebolt-Leopold J. S. Van Becelaere K. Hook K. Herrera R. (2003). Biomarker assays for phosphorylated MAP kinase. Their utility for measurement of MEK inhibition. Methods Mol. Med. 85, 31–38. 10.1385/1-59259-380-1:31 12710194

[B20] Solit D. B. Garraway L. A. Pratilas C. A. Sawai A. Getz G. Basso A. (2006). BRAF mutation predicts sensitivity to MEK inhibition. Nature 439 (7074), 358–362. 10.1038/nature04304 16273091 PMC3306236

[B21] Wortzel I. Seger R. (2011). The ERK cascade: Distinct functions within various subcellular organelles. Genes Cancer 2 (3), 195–209. 10.1177/1947601911407328 21779493 PMC3128630

[B22] Yoon S. Seger R. (2006). The extracellular signal-regulated kinase: Multiple substrates regulate diverse cellular functions. Growth factors. 24 (1), 21–44. 10.1080/02699050500284218 16393692

