# Supplementary material for: Establishment of a novel method to assess MEK1/2 inhibition in PBMCs for clinical drug development
Source: Front Cell Dev Biol. 2022 Dec 12;10:1063692. doi: 10.3389/fcell.2022.1063692 (PMC9790982; doi:10.3389/fcell.2022.1063692)
Supplement: Supplementary file 1 [file DataSheet1.PDF]

Supplementary Material

1 Supplementary Figures

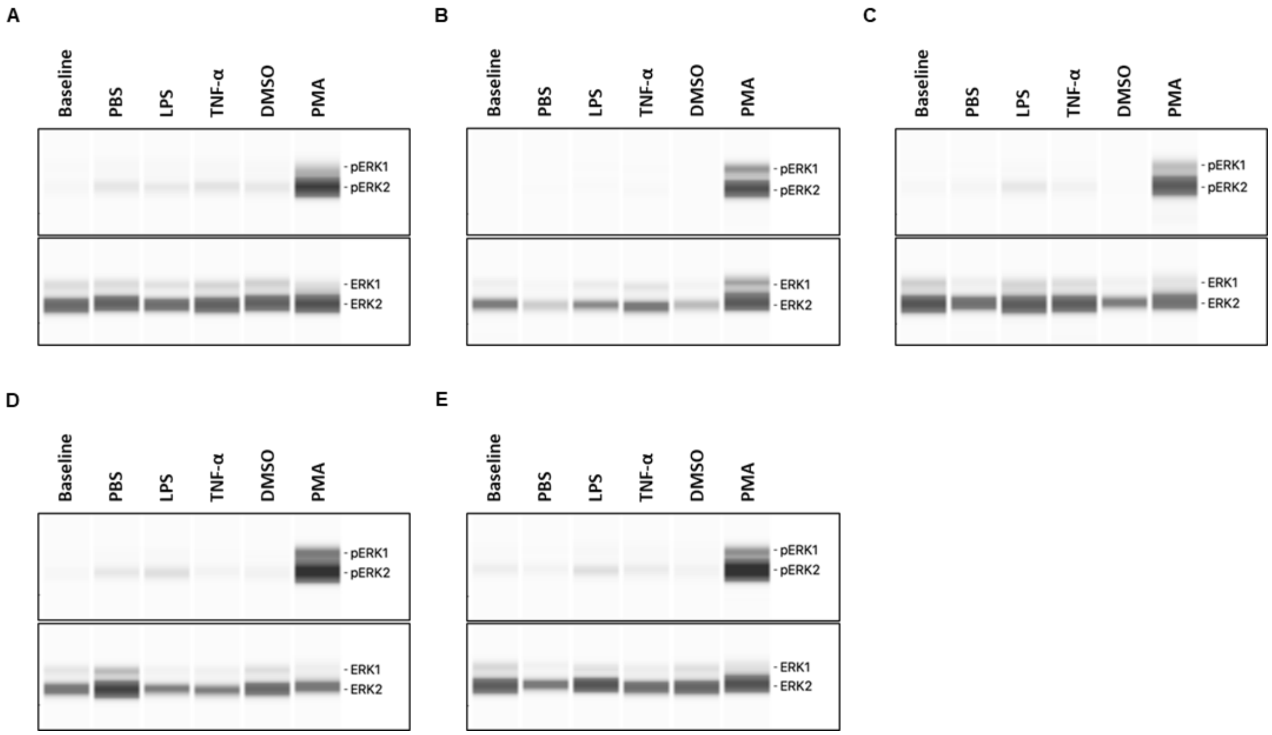

Supplementary Figure 1. Comparison of ERK1/2 phosphorylation levels in human PBMCs after stimulation with LPS, TNF-α, and PMA. PBMCs were stimulated with 1 µg/ml LPS, 20 ng/ml TNF-α, or 400 nM PMA. The cells were purified, lysed, and the levels of phosphorylated and total ERK1/2 were assessed with the Wes™ system. Data are presented as Western Blot-like bands. N = 5 donors (A-E).

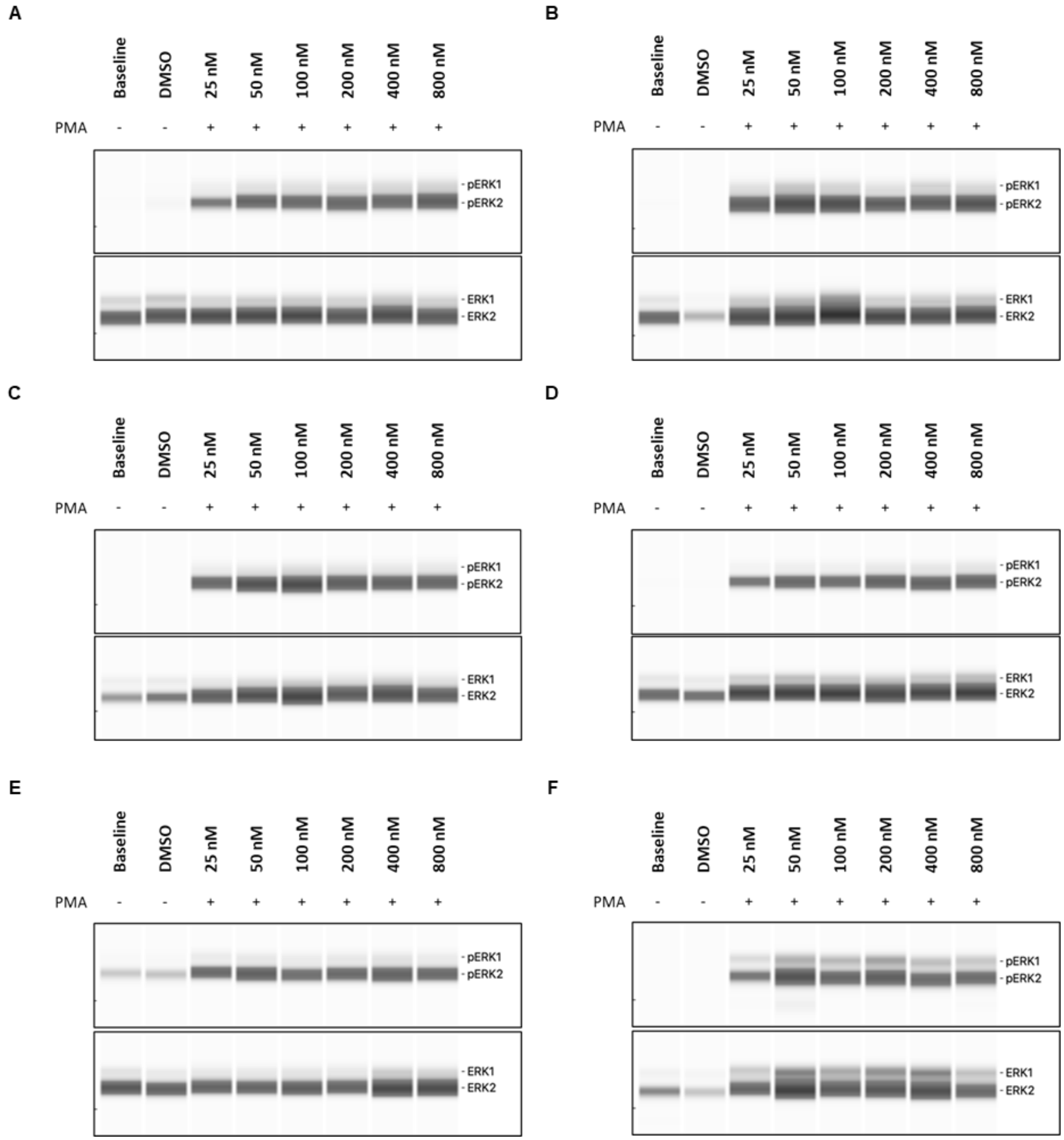

Supplementary Figure 2. PMA titration in human PBMCs. PBMCs were stimulated with 25-800 nM PMA for 30 min and the levels of phosphorylated and total ERK1/2 were assessed with the Wes™ system. Data are presented as Western Blot-like bands. N = 6 donors (A-F).

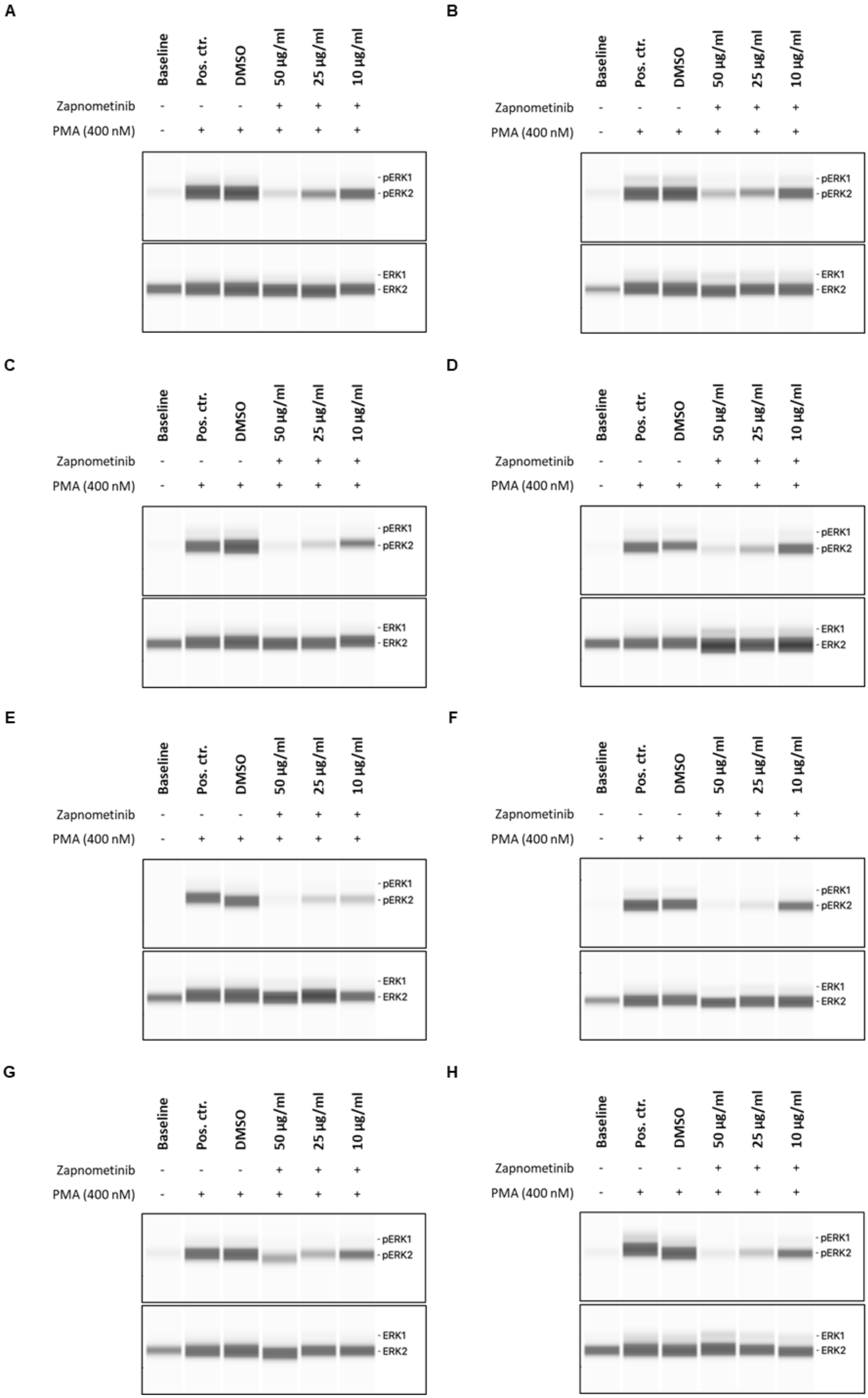
A
Baseline
Pos. ctr.
DMSO
50 µg/ml
25 µg/ml
10 µg/ml
Zapnometinib - - - + + +
PMA (400 nM) - + + + + +
pERK1
pERK2
ERK1
ERK2
B
Baseline
Pos. ctr.
DMSO
50 µg/ml
25 µg/ml
10 µg/ml
Zapnometinib - - - + + +
PMA (400 nM) - + + + + +
pERK1
pERK2
ERK1
ERK2
C
Baseline
Pos. ctr.
DMSO
50 µg/ml
25 µg/ml
10 µg/ml
Zapnometinib - - - + + +
PMA (400 nM) - + + + + +
pERK1
pERK2
ERK1
ERK2
D
Baseline
Pos. ctr.
DMSO
50 µg/ml
25 µg/ml
10 µg/ml
Zapnometinib - - - + + +
PMA (400 nM) - + + + + +
pERK1
pERK2
ERK1
ERK2
E
Baseline
Pos. ctr.
DMSO
50 µg/ml
25 µg/ml
10 µg/ml
Zapnometinib - - - + + +
PMA (400 nM) - + + + + +
pERK1
pERK2
ERK1
ERK2
F
Baseline
Pos. ctr.
DMSO
50 µg/ml
25 µg/ml
10 µg/ml
Zapnometinib - - - + + +
PMA (400 nM) - + + + + +
pERK1
pERK2
ERK1
ERK2
G
Baseline
Pos. ctr.
DMSO
50 µg/ml
25 µg/ml
10 µg/ml
Zapnometinib - - - + + +
PMA (400 nM) - + + + + +
pERK1
pERK2
ERK1
ERK2
H
Baseline
Pos. ctr.
DMSO
50 µg/ml
25 µg/ml
10 µg/ml
Zapnometinib - - - + + +
PMA (400 nM) - + + + + +
pERK1
pERK2
ERK1
ERK2

Supplementary Figure 3. Zapnometinib reduces ERK1/2 phosphorylation in human PBMCs. PBMCs were treated with 10, 25, and 50 µg/ml zapnometinib, followed by stimulation with PMA. Levels of phosphorylated and total ERK1/2 were assessed with the Wes™ system. Data are presented as Western Blot-like bands. N = 8 donors (A-H).

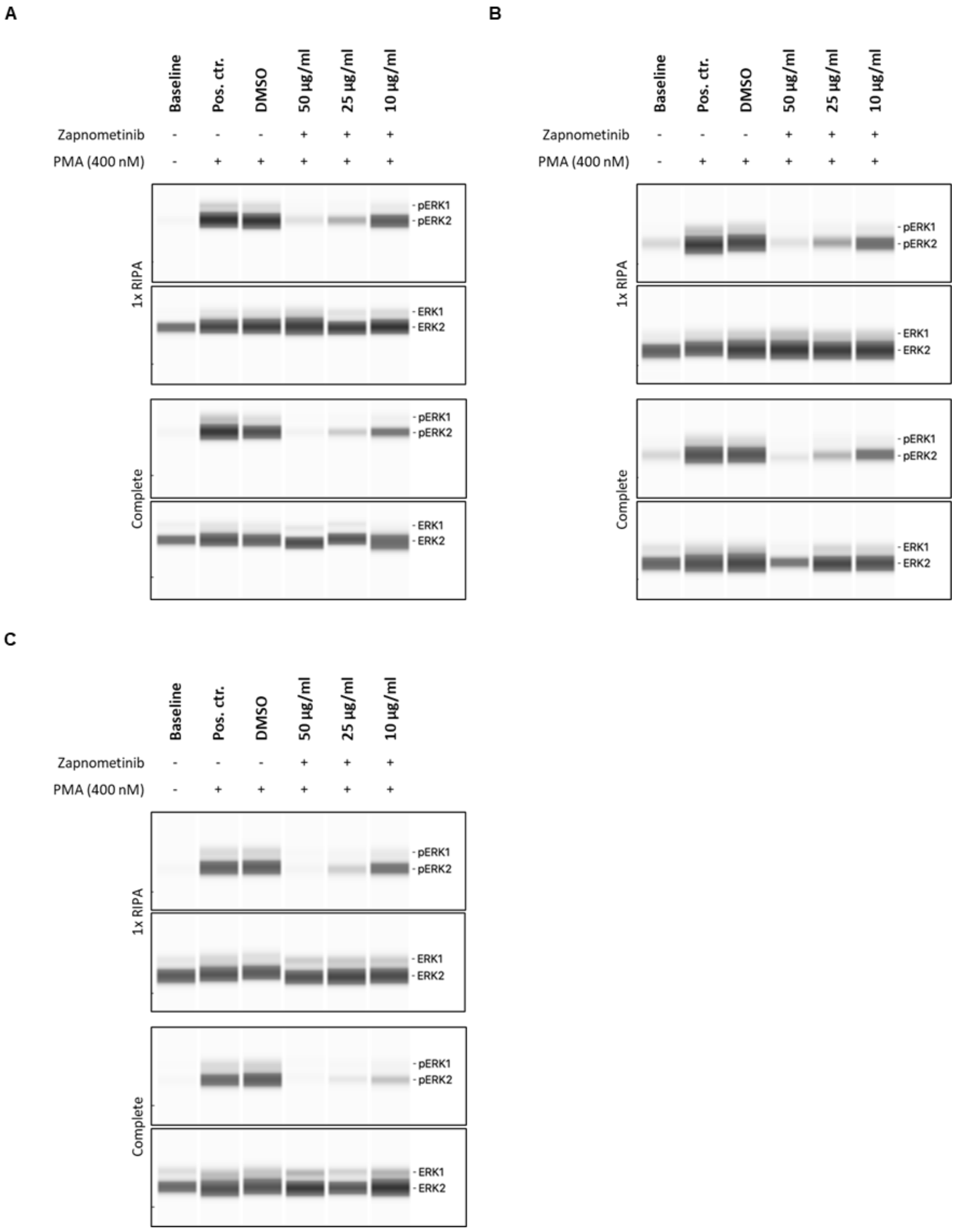

Supplementary Figure 4. Comparison of cell lysis using 1x RIPA or Complete lysis buffer. Cell treatment was performed as previously described and cells were lysed using either 1x RIPA (top panels) or Complete lysis buffer (bottom panels). Levels of phosphorylated and total ERK1/2 were assessed with the Wes™ system. Data are presented as Western Blot-like bands. N = 3 donors (A-C).

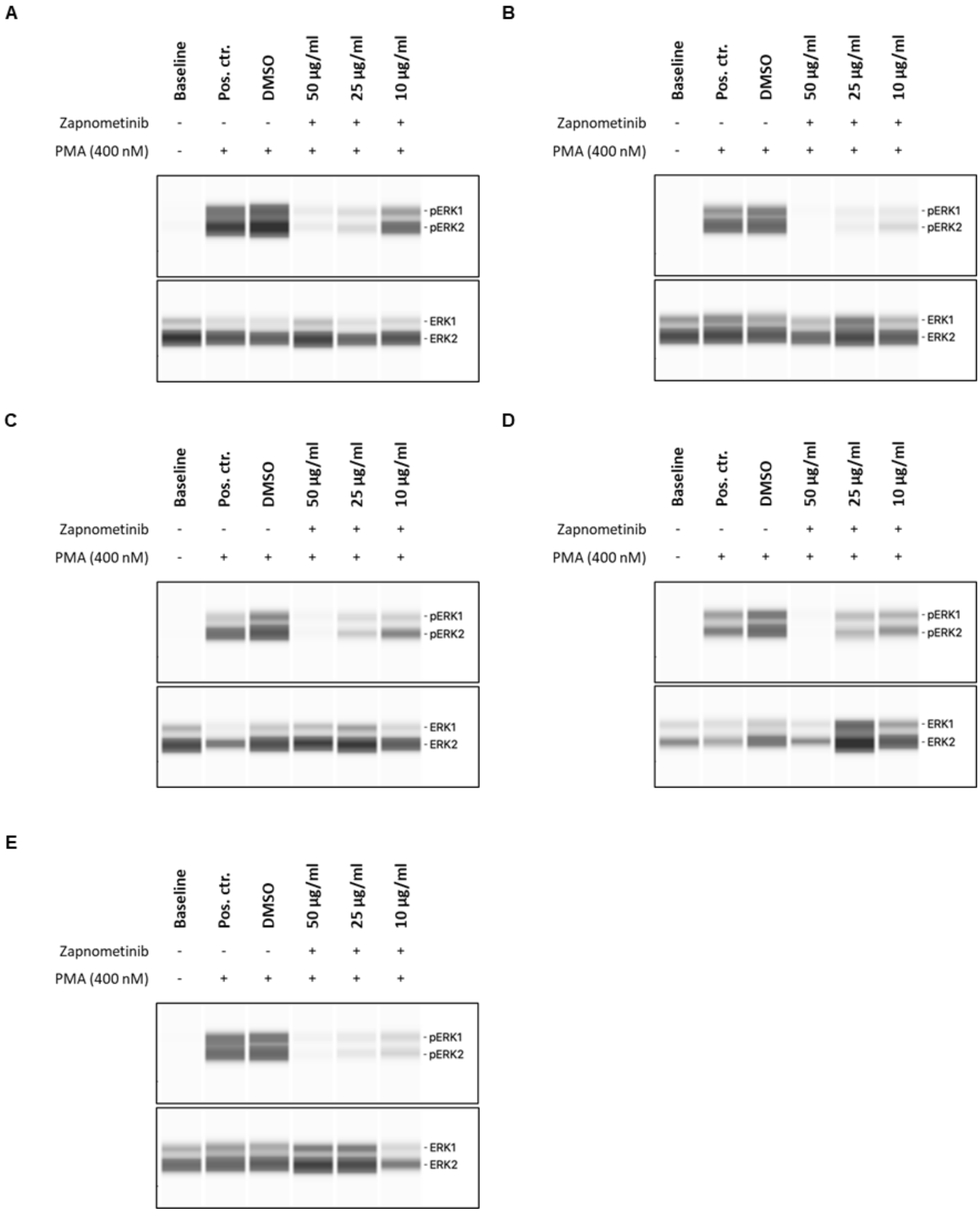

Supplementary Figure 5. Assessment of ERK1/2 phosphorylation levels with the Wes™ system. Cell treatment and lysis was performed as previously described and the samples were analyzed for phosphorylated and total ERK1/2 levels with the Wes™ system (in comparison to the MSD system; data shown in the main manuscript). N = 5 donors (A-E).
